# Network Diffusion Promotes the Integrative Analysis of Multiple Omics

**DOI:** 10.3389/fgene.2020.00106

**Published:** 2020-02-27

**Authors:** Noemi Di Nanni, Matteo Bersanelli, Luciano Milanesi, Ettore Mosca

**Affiliations:** ^1^Institute of Biomedical Technologies, National Research Council, Milan, Italy; ^2^Department of Industrial and Information Engineering, University of Pavia, Pavia, Italy; ^3^Department of Physics and Astronomy, University of Bologna, Bologna, Italy; ^4^National Institute of Nuclear Physics (INFN), Bologna, Italy

**Keywords:** integrative analysis, omics data, biological networks, precision medicine, network-diffusion

## Abstract

The development of integrative methods is one of the main challenges in bioinformatics. Network-based methods for the analysis of multiple gene-centered datasets take into account known and/or inferred relations between genes. In the last decades, the mathematical machinery of network diffusion—also referred to as network propagation—has been exploited in several network-based pipelines, thanks to its ability of amplifying association between genes that lie in network proximity. Indeed, network diffusion provides a quantitative estimation of network proximity between genes associated with one or more different data types, from simple binary vectors to real vectors. Therefore, this powerful data transformation method has also been increasingly used in integrative analyses of multiple collections of biological scores and/or one or more interaction networks. We present an overview of the state of the art of bioinformatics pipelines that use network diffusion processes for the integrative analysis of omics data. We discuss the fundamental ways in which network diffusion is exploited, open issues and potential developments in the field. Current trends suggest that network diffusion is a tool of broad utility in omics data analysis. It is reasonable to think that it will continue to be used and further refined as new data types arise (e.g. single cell datasets) and the identification of system-level patterns will be considered more and more important in omics data analysis.

## Introduction

“Omics” technologies provide data related to different types of molecular entities (e.g. DNAs, RNAs, proteins) at increasing sensitivity, down to single-cell level (Hu et al., 2018). This offers the opportunity for integrative analyses that lead to a more comprehensive view of a biological system (Higdon et al., 2015; Karczewski and Snyder, 2018). However, integrative analyses involve several issues due to types of biological information considered, coverage of the pool of molecular entities under investigation, data distribution types, noise and research questions that need to be addressed (Ritchie et al., 2015; Ahmad and Fröhlich, 2016; Huang et al., 2017), just to mention a few. Therefore, the development of integrative methods is one of the main challenges in bioinformatics.

**Figure 1:**
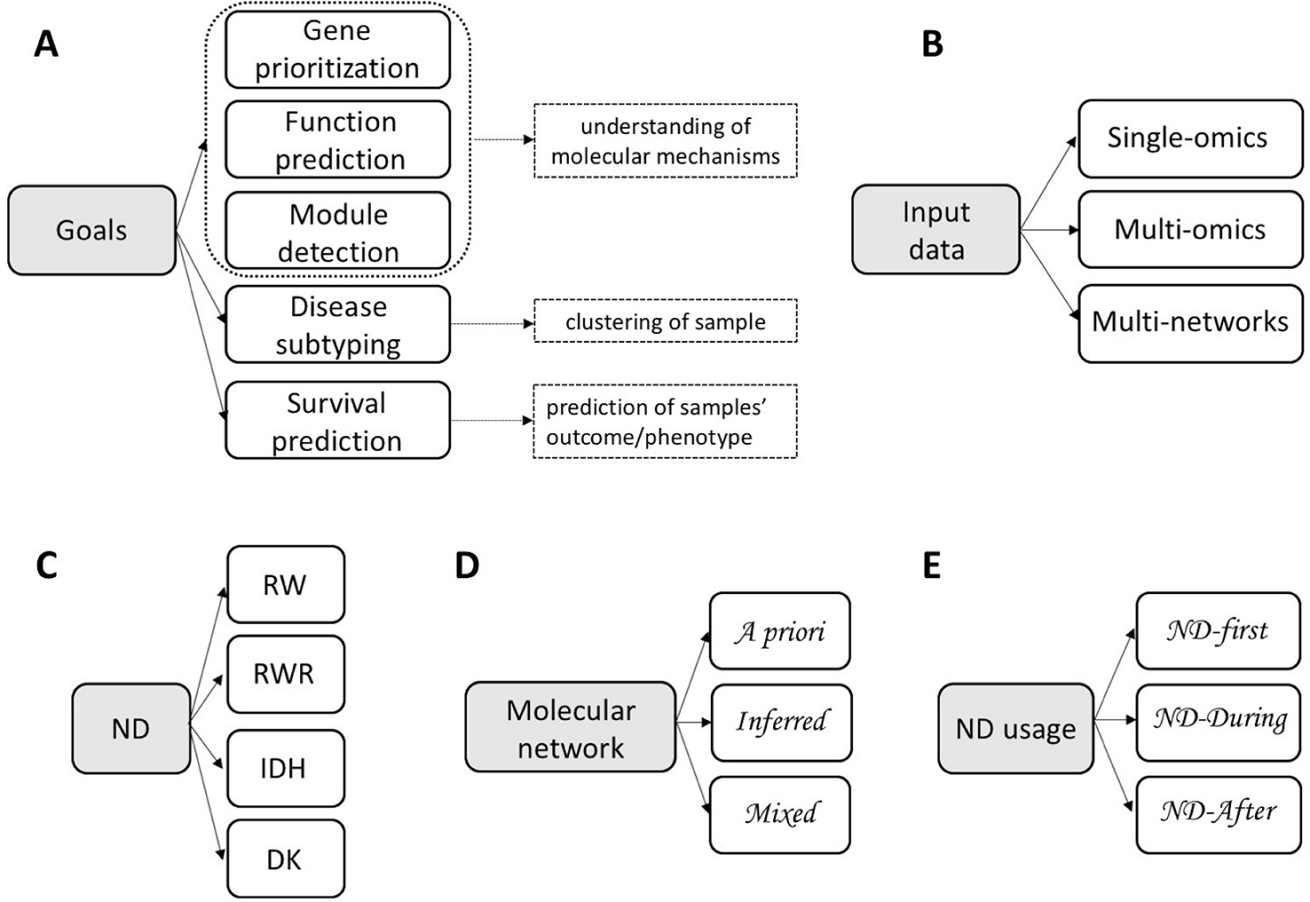
Classification of integrative methods. Criteria: **(A)** Goals; **(B)** Input data; **(C)** Network Diffusion (ND) model: Random Walk (RW), Random Walk with Restart (RWR), Insulated Heat Diffusion (IHD), Diffusion Kernel (DK); **(D)** Molecular network; **(E)** ND usage.

Integrative methods can be classified in three groups by objective (**Figure 1A**): understanding of the molecular mechanisms (e.g. genes prioritization, function prediction, module detection), clustering of samples (e.g. identification of disease subtypes) or prediction of samples' outcome/phenotype (e.g. survival) (Kristensen et al., 2014). These three objectives can be achieved using a single type or multiple types of omics, possibly combined with data about molecular networks (**Figure 1B**), in a supervised or unsupervised settings.

From a methodological point of view, the arising importance of interaction networks and the type of statistical approach pave the way for a first broad classification of integrative methods. In particular, these can be divided into four broad classes depending on whether they use molecular networks and Bayesian theory: network-free non-Bayesian, network-free Bayesian, network-based non-Bayesian and network-based Bayesian (Bersanelli et al., 2016a).

Molecular networks represent a powerful framework to integrate and explain omics datasets (Bersanelli et al., 2016a; Yan et al., 2017; Su et al., 2018). Indeed, the interactome, a term that designates the whole set of macromolecular interactions within a cell, could underlie most genotype to phenotype relationships (Vidal et al., 2011) and can be used to guide our understanding of how alterations detected by omics technologies perturb the system as a whole (Caldera et al., 2017). A known system-level pattern is, for instance, the presence of gene networks that are “hot” spots of mutations in cancer and reflect the several possible combinations of mutations that are likely to lead to the similar pathological phenotype, because affect the same pathways (Barabási et al., 2011; Boyle et al., 2017). More generally, network-based approaches enable the study of the relation between the topological and dynamical properties of a network and the biological system modelled by means of the network. For example, the distance between genes in a gene network is related to the functional similarity of the genes (Sharan et al., 2007) and their involvement in the same disease (Barabási et al., 2011). It is important to underline that network-based and network-free approaches to multi-omics data analysis can be combined within the same pipeline. For instance, a multivariate analysis for disease stratification can be performed using a (network free) classic multivariate regression and, then, the results can be further refined considering a network-based method.

In the last decades, the mathematical machinery of network diffusion (ND)—also referred to as network propagation—has been exploited in many network-based pipelines with different aims, like gene prioritization, gene module identification, drug target prediction and disease subtyping, thanks to its ability of amplifying association between variables (e.g. genes) that lie in network proximity (Cowen et al., 2017). Another important class of network-based approaches is the one inspired by percolation theory (Aleta and Moreno, 2019). One of the main applications of this theory is the study of robustness of biological systems, but also in finance and social networks (Reis et al., 2014; Brummitt and Kobayashi, 2015; Baggio et al., 2016; Gao et al., 2016).

ND has been incorporated in many pipelines that jointly analyse biological networks and multiple collections of scores (“layers”) derived from omics assays. These ND-based methods for multi-omics data analysis will be the main focus of this review.

The success of ND can be brought back to a series of benefits. Considering gene-centric datasets as a practical example, first of all, ND is a powerful way to embed the information about molecular interactions among genes into a gene-wise dataset. ND quantifies the proximity between genes in a global way, that is considering all possible paths among the genes, capturing the complexity of biological networks beyond the limits of local approaches (e.g. shortest path length) (Wang and Marcotte, 2010). ND highlights genes in network proximity and with high input scores. By so doing, it amplifies genetic associations according to the architecture of the molecular network, a result that offers insights in agreement with the so-called local hypothesis; that is, the hypothesis that genes that lie in network proximity within molecular networks co-work in the development of cellular functions and are therefore co-responsible for pathological phenotypes (Barabási et al., 2011). Moreover, by a data analysis perspective, ND transforms sparse input vectors into dense vectors. This operation eliminates missing values and ties, two situations that are often difficult to handle. This imputation step facilitates the joint analysis of different data types and is particularly important in the integration of multiple omics that vary in scope and coverage. For instance, mutations may affect just a few tens of genes of a tumor cell, while gene expression changes are observed for a much higher number of genes. More generally, in a multi-omic analysis of a biological process, only a subset of the genes is associated with the various types of measured alterations. In this context, ND can be used to highlight common network regions where different types of omics signals converge. ND can be performed not only at early stages of the integration pipeline (e.g. data imputation), but also at later stages, for instance to refine the results on the basis of molecular network data. Lastly, ND is suitable to analyse patient-level molecular profiles, promoting studies within the scope of precision medicine.

Recently, Cowen et al. (2017) provided a general overview of the unifying mathematical machinery of ND, showing its broad utility in several problems of genetic research, while, previously, Wang and Marcotte (2010) described the application of ND to the problem of predicting gene function and phenotype. Here, we focus on the use of ND in multi-omics data analysis. We extend previous studies by adding recent developments in the field. We will give hints on molecular networks, the scaffolding on which ND takes place. Then, we will summarize the mathematical machinery of ND. Lastly, we will review the integrative methods that use ND by aim, input data type, molecular network, way in which ND is exploited during the integrative analysis and application. The trends of recent works suggest that ND will continue to be used and further developed to meet the requirements of novel research questions that arise as novel data types will be more and more available, like single cell datasets.

## MoleculaR Networks: The Scaffolding for Diffusion

Network-based methods require, by definition, a molecular network that enters the analysis pipeline at some point. The complex web of molecular interactions that occur within human cells is often referred to as “interactome” (Barabási et al., 2011). Such interactions can be of rather different types and are usually distinguished in two classes: *biophysical* and *functional* (Caldera et al., 2017). Biophysical interactions indicate actual molecular contact between two molecular entities, such as protein–DNA biding or protein–protein binding in a protein complex. Functional interactions indicate any kind of biologically relevant interaction (at the molecular scale), like co-expression or synthetic lethality.

There is still no unique reference for the human interactome (Luck et al., 2017), but several efforts are underway. Four proteome-scale PPI interaction maps have been recently generated using different high-throughput approaches based on binary interaction or complex mapping (Luck et al., 2017). The Genotype-Tissue Expression (GTEx) project aims at the construction of a specific network for each major human tissue (GTEx Consortium, 2015). Projects like ENCODE97 and the Roadmap Epigenomics provide data about gene regulatory networks (Kellis et al., 2014; Kundaje et al., 2015). The IMEx Consortium is an international collaboration of major public interaction data providers aimed at establishing a non-redundant set of biophysical molecular interactions (Orchard et al., 2012). In addition to *primary databases*, which collect curated experimental data from small and/or large scale studies, there are several *meta-databases*, which integrate data from several primary databases, and *prediction-databases*, which also provide predicted (biophysical and/or functional) interactions obtained from the analysis of biological datasets (De Las Rivas and Fontanillo, 2010).

Multiple collections of scores can be mapped on molecular networks in rather different ways, depending on data types and data analysis purposes. We can classify the resulting networks in three broad categories: multi-weighted networks, multiplex networks and networks of networks.

In a multi-weighted network, a series of weights are associated with nodes and/or links. For instance, the same biological network can be characterized by different omics weights on different layers (e.g. gene expression, methylation, somatic mutations). A multi-weighted network therefore consists of a single-layer network with multiple attributes associated with the same nodes and links, but sometimes can be referred to as a multi-layer network.

Two categories of structural multi-layer networks are multiplex networks and networks of networks. A multiplex is a collection of networks with the same set of nodes and varying intra-layer topologies and inter-layer relationships are trivially given (Menichetti et al., 2014).

A network of networks (sometimes also referred to as heterogeneous networks) is a collection of networks with different nodes (in principle also representing entities of different nature) with multiple types of connections (specific intra-layer links and specific inter-layer connections) (Kivelä et al., 2014). The classification of multi-layer networks is indeed non-trivial; for instance, the categories described can have significant overlaps. It is possible to build hybrid networks where on a core multiplex some layer-specific nodes and links are introduced and consequently different types of inter-layer links are established; for more details about multilayer networks and their classification see the work of Kivelä et al. (2014).

## Mathematics of the Network Diffusion Process

ND processes can be summarized as the spreading of biological information throughout the network along network edges, initially retained in the so-called “seed nodes”. Each node will therefore gain or lose biological information according to the network proximity to the seeds and to its topological features. ND is realized by means of different methods that can be brought back to random walks, random walks with restarts and diffusion kernels.

From a mathematical perspective, considering a network *G* of *n* nodes, the biological information is encoded in an *n*-dimensional array ***x***_0_ where the *i*-th entry accounts for the amount of biological signal initially present in node *i*. We can therefore define ***x***_0_ as the initial state of the network. Then, starting from *t*=0 up to a fixed time (finite or infinite) the state of the network ***x****_t_* evolves according to the network topology until it reaches a final state ***x***_T_, where, as previously mentioned, *T* can either a finite or an infinite time. Under the appropriate settings, when *T*=∞, the final state of the diffusive algorithm may correspond to a steady state or steady-flow state of an associated physical model, allowing a clear interpretation of the results (Bersanelli et al., 2016b).

In general, the final state of a diffusion process consists of a graph-based transformation *f_G_* of the initial biological information ***x***_0_, which is linear in most cases so that *f_G_* reduces to a matrix ***M****_G_* and

(1) $$x_{T}=f_{G}\left(x_{0}\right)=M_{G}\cdot x_{0}$$

We classify the diffusion processes used by integrative methods, similarly to Cowen et al. (2017), on the basis of the specific transformation ***M****_G_* in four categories (**Table 1** and **Figure 1C**):

1. Random Walk (RW): $M_{G}={\left[AD^{-1}\right]}^{k}$;
2. Random Walk with Restart (RWR): $M_{G}=\alpha {\left[I-\left(1-\alpha \right)D^{-1/2}AD^{-1/2}\right]}^{-1}$;
3. Insulated Heat Diffusion (IHD): $M_{G}=\alpha {\left[I-\left(1-\alpha \right)AD^{-1}\right]}^{-1}$;
4. Diffusion Kernel (DK): ***M***_*G*_ =  *e*^*α*(***D***−***A***)^.

**Table 1:** Network diffusion based methods for the integrative analyses of multiple biological layers.

Method	Input	Integration Level	ND		Network	Goal	Language and Availability	URLs
			Type	Application				
Dmfind (Bersanelli et al., 2016b)	gene mutations	single omics	RWR	ND-first	A priori	module detection	R package for download	https://www.itb.cnr.it/web/bioinformatics/dmfind
EMDN (Ma et al., 2017a)	DNA methylation, gene expression	multiple omics	RWR	ND-first	Inferred	module detection	R package for download	https://github.com/william0701/EMDN
EPU (Yang et al., 2014)	gene expression, PPI, gene ontology, gene-phenotype association data and phenotype similarity network	multiple networks	RWR	ND-first	Mixed	gene prioritization	–	–
GeneMANIA (Mostafavi et al., 2008)	co-expression, PPI, genetic interaction, co-localization, shared protein domains	multiple networks	RWR	ND-first	A priori	function prediciton	Web server	http://apps.cytoscape.org/apps/genemania
Mashup (Cho et al., 2016)	PPI	multiple networks	RWR	ND-first	A priori	function prediciton	Matlab code for download	http://cb.csail.mit.edu/cb/mashup/
M – module (Ma et al., 2014)	gene mutation, gene expression	multiple omics	RWR	ND-first	Inferred	module detection	R package for download	http://tanlab4generegulation.org/software/
mND (Di Nanni et al., 2020)	gene mutation, gene expression	single omics, multiple omics	RWR	ND-first	A priori	gene prioritization	R package for download	https://www.itb.cnr.it/web/bioinformatics/mnd
NetBag (Wu et al., 2015)	gene expression	single omics	RWR	ND-first	A priori	disease subtyping	–	–
NetICS (Dimitrakopoulos et al., 2018)	aberration events, gene expression	multiple omics	IHM	ND-first	A priori	gene prioritization	Matlab code for download	https://github.com/cbg-ethz/netics
NBS (Hofree et al., 2013)	gene mutations	single omics	RWR	ND-first	A priori	disease subtyping	Matlab code for download	http://chianti.ucsd.edu/~mhofree/NBS/
NBS2 (Zhang et al., 2018)	gene mutations	single omics	RWR	ND-first	Mixed	disease subtyping	Phyton package for download	https://github.com/wzhang1984/NBSS
RegNet (Seifert and Beyer, 2017)	CNV, gene expression	multiple omics	RW	ND-after	Inferred	gene prioritization	R package for download	https://github.com/seifemi/regNet
Ruffalo et al. (2015)	gene mutations, gene expression	multiple omics	RWR	ND-first	A priori	gene prioritization	–	–
Shi et al. (2016)	gene mutations, gene expression	multiple omics	RW	ND-first	Mixed	gene prioritization	–	–
SRF (Le Van et al., 2016)	gene mutations, gene expression	multiple omics	RWR	ND-first	A priori	disease subtyping	Java code for download	https://github.com/rankmatrixfactorisation/SRF
SNF (Wang et al., 2014)	DNA methylation, gene expression	multiple omics	DK	ND-during	Inferred	survival prediction, disease subtyping	R and Matlab code for downloads	http://compbio.cs.toronto.edu/SNF/SNF/Software.html
stSVM (Cun and Fröhlich, 2013)	gene expression (mRNA, miRNA)	multiple omics	DK	ND-after	A priori	gene prioritization, survival prediction	R package for download	https://www.rdocumentation.org/packages/netClass/versions/1.2.1
TieDie (Paull et al., 2013)	gene mutations, gene expression	multiple omics	IHM	ND-first	A priori	module detection	Python and Matlab code for downloads	https://sysbiowiki.soe.ucsc.edu/tiedie
WSNF (Xu et al., 2016)	gene expression (mRNA, miRNA)	multiple omics	DK	ND-during	Inferred	survival prediction, disease subtyping	R package for download	http://nugget.unisa.edu.au/Thuc/cancersubtypes

RWR, random walk with restart; DK, diffusion kernel; IHM, insulated heat model; RW, random walk; CNV, copy number variations; PPI, Protein-Protein interaction network.

Here above, ***A*** is the adjacency matrix of the network, ***D*** is a diagonal matrix of nodes degree (number of interactions), *k* is the number of time-steps and *α*∈(0,1) is a tuning parameter. Differently from Cowen et al. (2017) we choose to differentiate between RWR and IHD. In fact, the different normalization of the adjacency matrix ***A*** (symmetric for the RWR, column normalization for the IHD) implies different behaviours in the relative diffusion processes. Indeed, the RWR implies a symmetric diffusion where information flows through each link with the same intensity in each direction (Vanunu et al., 2010). Conversely, IHD implies an asymmetric diffusion where information (or heat) tends to flow out from highly connected nodes much easier than from poorly connected ones (Leiserson et al., 2015). Such differences in the diffusion matrix therefore imply dissimilar behaviours of information flow, mainly in relation to network hubs: at infinite time in the RWR hubs tend to naturally gather relatively more information than in the IHD, since IHD is characterized by an intrinsic hub penalization. Therefore, despite RWR and IHD are conceptually similar, they may present sensibly different results, especially when applied to complex biological networks with thousands of vertices and tens to hundreds thousands links.

Independently from the specific kind of diffusion model, the matrix ***M***_G_ is usually hard to recover analytically because it implies inverting or power-expanding a high-dimensional graph-based transition matrix: alternative numerical approaches would be needed and the direct inversion of the matrix ***M***_G_ is possibly replaced with converging iterative procedures (Zhou et al., 2004).

The choice of the most appropriate diffusion process depends on the goal of the analysis. For instance, if one is interested only in considering a local neighborhood of the seeds may choose RW with a finite number of steps (Cun and Fröhlich, 2013), while RWR and IHD quantify network proximity to seeds considering simultaneously all the possible network paths among network nodes (Hofree et al., 2013; Leiserson et al., 2015).

## Network Diffusion in Integrative Data Analysis

ND requires data about the variables (***x***_0_) and about their relations (***A***). An important difference between integrative methods that use ND concerns the type of network in use, that is the way in which the adjacency matrix is defined. Three broad categories can be recognized (**Table 1** and **Figure 1D**): the topology of the network in use can be defined by means of *a priori* knowledge, e.g. collected from molecular interactions databases; alternatively, a network can be *inferred* from the analysis of one or more biological datasets; lastly, a mixed approach that combines *a priori* and novel knowledge is possible.

**Figure 2:**
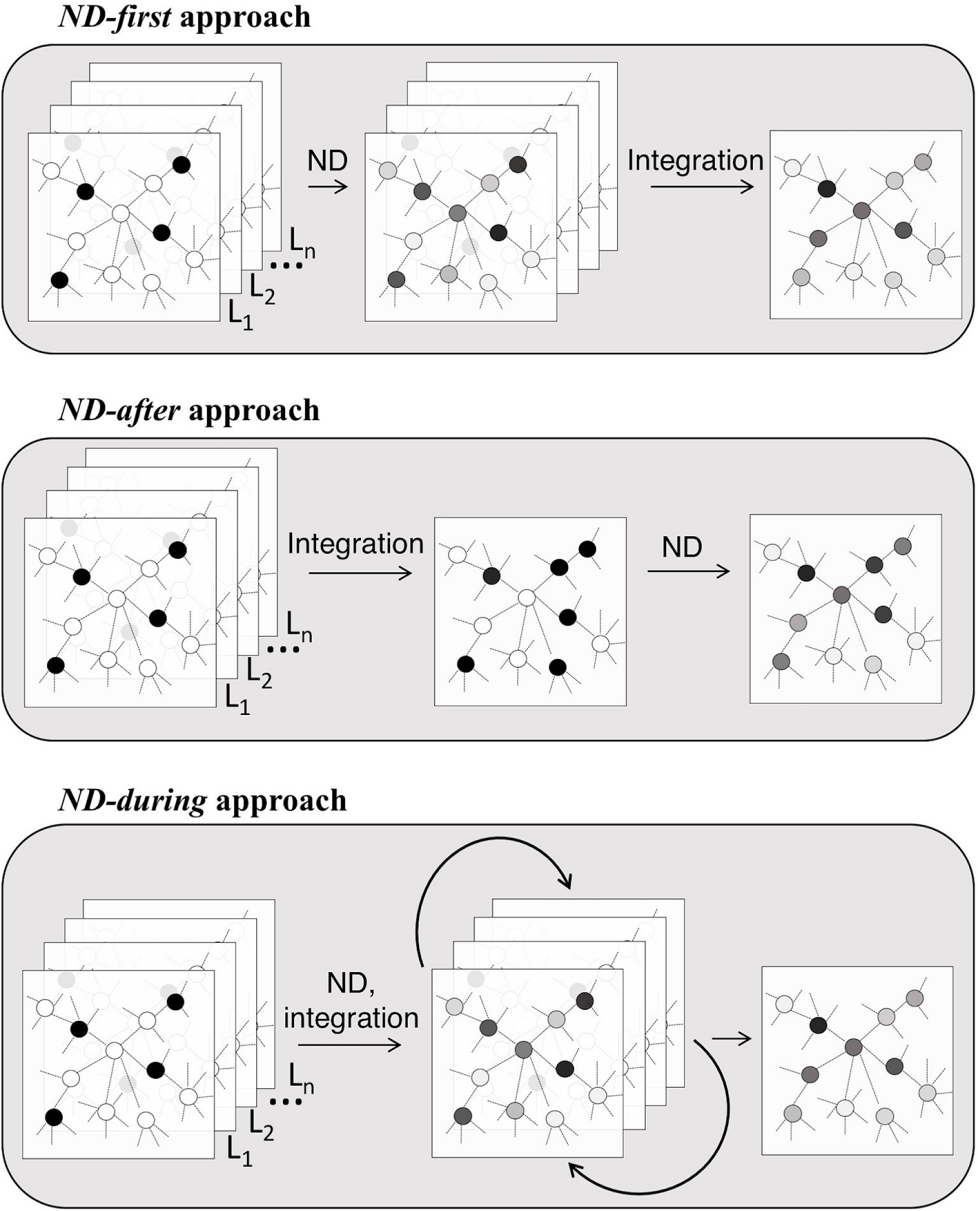
Ways in which ND enters the integrative analysis pipelines.

ND can be applied before, after or during the “integration step” of the analysis pipeline (**Table 1** and **Figures 1E**, **2**). In the *ND-first* approach, ND is applied to a series of collections of initial scores, each of which summarizes data of a single sample or multiple samples; the resulting collections of ND scores are subsequently integrated. An example of this approach is TieDIE (Paull et al., 2013), where ND is applied to two collections of scores, one representing mutated genes while the other differentially expressed genes, on the same network; the two resulting ND score vectors are then jointly analysed and the minimum of the two ND scores of a gene is considered as the one chosen for the gene.

The *ND-after* approach consists in the application of ND after a first process of integration of different data types into a unique structure. For instance, stSVM (Cun and Fröhlich, 2013) first integrates omics data and subsequently applies ND to define a global ranking of miRNA and mRNA using statistics about their differential expression integrated in a heterogeneous network.

The *ND-during* refers to the application of a type of ND in which each layer communicate information one another. This is the case of SNF (Wang et al., 2014), in which patient similarity networks, obtained from each of their data types separately, exchange information during the ND process, leading to a unique “fused” patient network.

**Figure 3:**
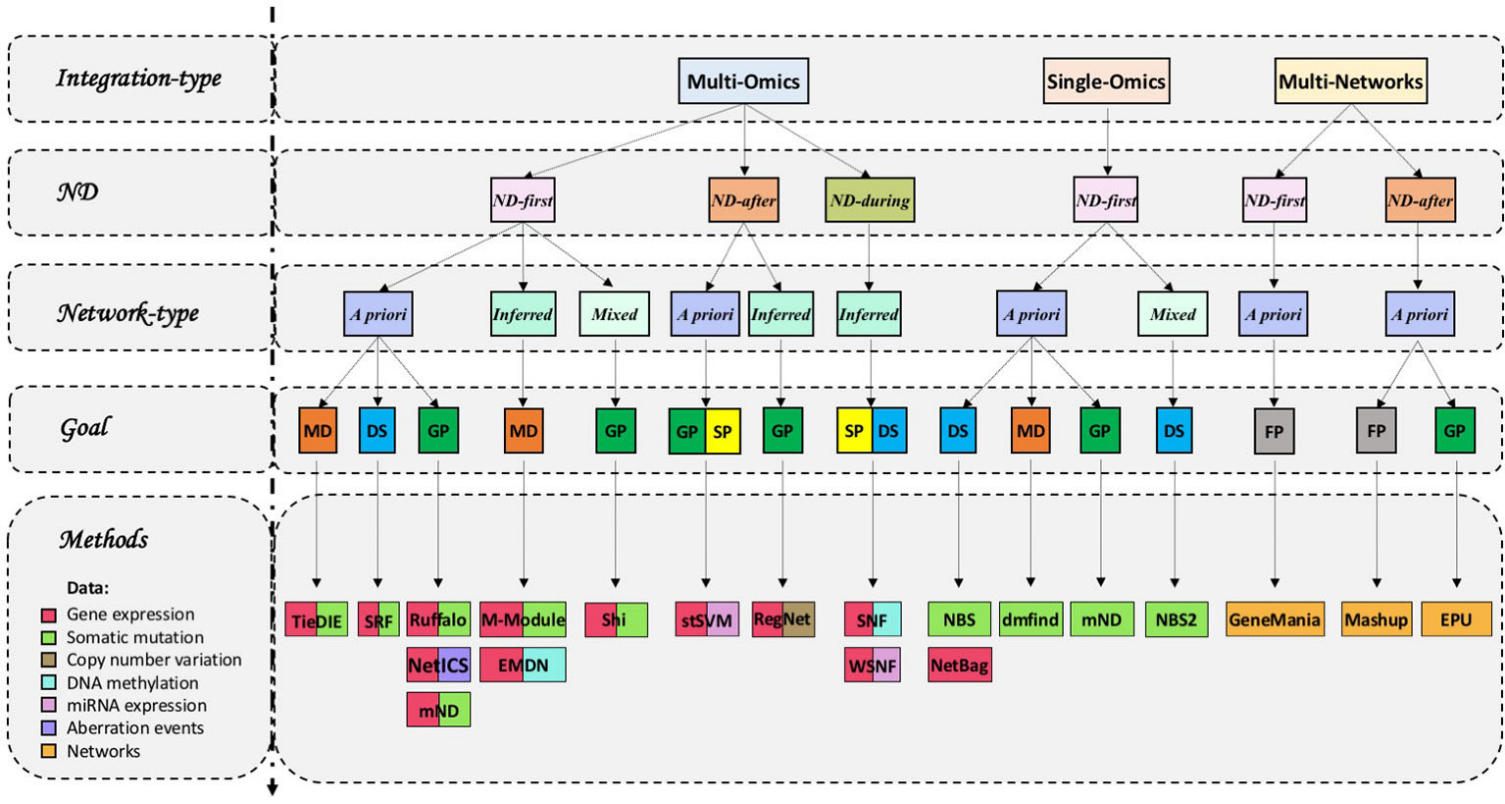
Network diffusion methods for the integrative analyses of multiple biological layers. GP, gene prioritization; MD, module detection; FP, function prediction; DS, disease subtyping; SP, survival prediction. We classified methods according to their main use described by the respective authors.

On the basis of data types, we can distinguish integrative methods that use ND to analyse a single type of omics, multiple omics or multiple networks (**Table 1** and **Figures 1B**, **3**).

### Single Omics

Integrative methods for the analysis of a single type of omics consider a series of molecular profiles, such as patient-wise mutation profiles.

The method called “dmfind” (Bersanelli et al., 2016b) compares ND scores obtained from a series of descriptive statistics, such as gene mutation frequencies. Subsequently, the network smoothing index (NSI) is obtained by comparison of ND scores with initial molecular profiles (Bersanelli et al., 2016b). When applied to gene networks, NSI highlights genes in network proximity enriched by initial information according to a tuning parameter *ϵ* (Barabási et al., 2011). The integration is therefore realised by subtracting NSIs belonging to two patient groups (ND-first), an operation that prioritizes genes that participate in differentially enriched modules (Bersanelli et al., 2016b).

tlsb 0.1ptNBS (Network-Based Stratification) (Hofree et al., 2013) is a method that stratifies tumor mutations finding clusters of similar patients. It applies ND to a binary somatic mutation matrix (genes-by-samples). Then, the resulting collections of ND scores are jointly analysed (ND-first) using a network-constrained non-negative matrix factorization to find *k* patient groups. It has been applied to study 13 cancer types with exome-level mutation data (Zhong et al., 2015), liver cancer (Fujimoto et al., 2016) and in a pan-cancer genomic analysis (Liu and Zhang, 2015).

NetBags (NETwork Based clustering Approach with Gene signatures) (Wu et al., 2015) essentially applies the strategy of NBS to a binary genes-by-samples matrix that represents the significantly expressed genes.

NBS^2^ (Network-Based Supervised Stratification) (Zhang et al., 2018) was proposed as a development of NBS (Hofree et al., 2013). NBS^2^ uses cancer-specific *a priori* knowledge of molecular interaction networks. Unlike previous approaches, the weights of each molecular interaction are adjusted by a supervised strategy so that the stratification of propagated mutation profiles after random walk is close to the pre-defined tumor subtypes.

Lastly, mND (Di Nanni et al., 2020) has been developed to yield gene prioritizations and it is applicable to both datasets originating from a single type or multiple types of omics, which can be distributed over multiple layers with the same underlying network structure. We will further describe this method in the next section.

### Multi-Omics Integration

In multi-omics data integration each layer typically contains scores obtained from a distinct omic assay. Most methods deal with two types of layers (**Figure 3**).

#### Genomics and Transcriptomics

Many methods tackled the problem of analysing the relation between genomic aberrations and gene expression changes.

Ruffalo et al. (2015) presents a ND-based method to predict “silent” players in cancer by integration of somatic mutations and gene expression data, where a silent player is a gene neither mutated nor differentially expressed but which plays a role in cancer development and progression. Inputs are represented as two binary matrices of somatic mutation and gene expression (genes-by-samples).

The authors explored several ways (e.g. the minimum, the maximum, the product, the average) of combining diffusion scores (ND-first) to obtain the features of a logistic regression model that predicts a gene's association with cancer.

Also Shi et al. (2016) use patient-wise gene mutation and gene expression data to prioritize genes. The approach constructs a bipartite graph of outlying genes and mutated genes considering an influence graph (that captures *a priori* biological pathway information), mutational and expression data. A two-step diffusion is performed to calculate diffusion scores for each patient and these scores are subsequently combined (ND-first) by robust rank aggregation.

mND (Di Nanni et al., 2020) prioritizes genes taking into account the network proximity of the genes and their first neighbours to other altered genes considering multiple types of biological evidence. It works without constraints for the type and number of input, applying ND to a general gene-by-samples matrix, where each column represents a vector of scores (e.g. gene mutation frequencies, p-values from differentially expressed genes). Then, the resulting collections of ND scores are integrated (ND-first) by calculating for each gene the product between the sum of its network constrained scores and the sum of the contributions of its top *k* first neighbours. Beyond prioritizing genes, mND provides the opportunity to classify genes in each layer suggesting genes role in relation to the context of the alterations detected.

Differently from the methods described above that yield gene prioritizations, TieDIE (Tied Diffusion Through Interacting Events) (Paull et al., 2013) has been developed to identify a subnetwork that links a source gene set (S) carrying genomic alterations to a target set (T) of differentially expressed genes on the same *a priori* network. TieDIE transforms the two collections of input scores in the corresponding ND scores and then (ND-first) the minimum of the two scores of a gene is used as the final score for that gene. TieDIE has been used to study several cancers, such as, Papillary Thyroid Carcinoma (Agrawal et al., 2014), Prostate Cancer (Drake et al., 2016), Leukemia (Huang et al., 2018) and in an extensive immugenomic analysis of 33 diverse cancer types (Thorsson et al., 2018).

Another method that seeks to identify gene modules is M-Module (Ma et al., 2014). It infers co-expression networks from multiple data that represent disease stage transitions. Then genes are ranked in each networks *via* ND, incorporating also gene mutations as priors. In each network, ND scores are transformed in gene ranks, gene ranks into z-scores and the average z-score across all is used to obtain a final gene rank (ND-first). Gene modules are therefore identified using a graph entropy-based measure that quantifies connectivity of a module in multiple networks. Authors of M-Module proposed different variants of the algorithms: NMF-DM, in which modules of each network are discovered using a non-negative matrix factorization algorithm (Ma et al., 2016), SMMN, which uses modularity measure to discovery modules (Ma et al., 2017b) and S2-jNMF a novel semisupervised joint nonnegative matrix factorization algorithm (Ma et al., 2018). M-Module has been applied to several studies [e.g. Chen et al. (2016); Han et al. (2017); Zhou et al. (2017)].

SRF (Le Van et al., 2016) aims at discovering cancer subtypes by combining mutation and expression data across samples. ND is applied only to the binary matrix of gene mutations. The identification of subtypes is performed by rank matrix factorization of the ranked diffusion matrix and ranked expression matrix (ND-first).

Copy number variations (CNVs) are another type of genomics aberration that has been jointly analysed with transcriptomics. The main goal of RegNet (Seifert and Beyer, 2017) is the quantification of the impact of gene expression changes on user-defined target genes in a network inferred from gene expression and CNVs. The approach learns a regulatory network by modelling the expression level of each gene as a linear combination of the expression levels of all other potential regulator genes and the gene-specific copy number, lasso regression is used in combination with a significance test for lasso (Lockhart et al., 2014) to find the relevant predictors for each gene. Next, ND is applied using the learned network to quantify impacts of sample-specific gene expression changes on other clinically relevant target genes using network-diffusion. RegNet was able to predicts novel cancer gene candidates in oligodendrogliomas (Gladitz et al., 2018).

#### Epigenomics and Transcriptomics

The algorithm of M-Module is employed in EMDN framework (Epigenetic Module based on Differential Networks) (Ma et al., 2017a) to characterize epigenetic modules by using differential co-methylation and co-expression networks, without incorporating genes mutations information as prior information. In this way EMDN applies ND as RW without restart, but with a symmetric normalization of the adjacency matrix.

An interesting method that aims to find disease subtypes and predict phenotypes is SNF (Similarity Network Fusion) (Wang et al., 2014). It works without constraints for the type of input but requires that samples are matched across omics. First, networks of samples for the various types of omics are built, then, networks are fused into one network by using the non-linear method of message passing theory (KNN and graph diffusion) that iteratively updates each of the network making it more similar to other networks in each step.

Several studies in cancer have exploited SNF method to integrate GE and DM data, like: Kidney Renal Cell Carcinoma (Deng et al., 2016), medulloblastoma (Cavalli et al., 2017); further, thanks to its versatility, SNF has been used to integrate other types of omics: miRNA and GE in Colorectal liver metastasis (Pitroda et al., 2018) and in Ovarian cancer (Zhang et al., 2016); miRNA, mRNA, lncRNA, and DNA methylation in Pancreatic Ductal Adenocarcinoma (Raphael et al., 2017); GE, miRNA and CNV in triple-negative breast cancer (Chiu et al., 2018).

#### Transcriptomics: mRNA and miRNA

Xu et al. (2016) have proposed a modification of SNF method called WSNF (Weighted Similarity Network Fusion) that takes into consideration the level of importance of genes to identify disease subtypes. WSNF constructs a miRNA-TF-mRNA regulatory network from different interaction databases, then assesses the weight of each features (miRNA, TF, mRNA), calculated as a linear combination of two terms: ranking of features obtained using ND and expression variation across all patients in expression datasets. Weights are introduced into the formula of Euclidean distance to calculate the distance between two patients then SNF method is applied.

stSVM (smoothed *t*-statistic support vector machine) (Cun and Fröhlich, 2013) combines *a priori* network information and omics data (miRNA and GE) to discover biomarker signature and predict disease prognosis. It smoothens gene-wise statistics from experimental data (both miRNA and gene expression) over the biological network, constructed by integration of PPI with miRNA-target gene network, using a *P*-step random walk kernels. A permutation test is conducted to select significant genes that will be used to train a support vector machine (SVM) classifier. It has been used in an integrative study of miRNA and GE to predict response to a monoclonal antibody in Head and Neck Squamous Cell Cancer (De Cecco et al., 2017).

#### Genomics, Epigenomics and Transcriptomics

NetICS (Network-based Integration of Multi-omics Data) (Dimitrakopoulos et al., 2018) prioritizes cancer genes by their mediator effect, defined as the proximity of the gene to aberration events (SM, CNV, DM, a differentially expressed miRNA), differentially expressed genes and proteins in a molecular network given *a priori*. The method uses a per-sample bidirectional IHD process and initial heat vectors (***h***_1_, ***h***_2_) are defined, respectively, as the number of the aberrant and differentially expressed genes of the sample. Final scores for all genes are obtained by means of the Hadamard product of the exchanged heat matrices (***E***_1_, ***E***_2_) (ND-first): ***E*** = ***E***_1_ ◦ ***E***_2_.

Lastly, diffusion scores of all samples are combined to obtain global gene ranking *via* a robust aggregation, in which a gene's rank is calculated as the sum of its per-sample ranks.

### Integration of Multiple Networks

In the integration of multiple networks each layer represents a biological network. The two main applications are gene function prediction and gene prioritization.

Mashup (Cho et al., 2016) uses ND on several protein–protein interaction networks to predict gene function and genetic interactions. It applies RWR algorithm separately on each network and then a matrix factorization based technique is used to reduce dimension of the diffusion results (ND-first). The feature learning step allows to obtain a low-dimensional feature vectors of proteins that best approximates the RWR matrix and results more robust to noise; feature vectors are used to train SVM classifiers to predict genetic interactions.

Mostafavi et al. (2008) developed GeneMANIA (Multiple Association Network Integration Algorithm), a tool for predicting gene function by integration of multiple networks (e.g. co-expression, PPI, genetic interaction, co-localization, shared protein domains). Given *d* networks encoded as matrices ***W**_1_*,…,***W**_d_*, they are integrated into a “composite network” (***W***^comb^), obtained by weighted average of individual networks:

$$W^{comb}=\underset{h}{\sum }\alpha_{h}W_{h}$$

where the vector [***α*** = *α*_1_, …, *α_d_*] corresponds to network weights and is computed by solving a ridge regression problem. Then given the ***W***^comb^ matrix, a variation of the Gaussian field label propagation algorithm (a RWR where functions of unlabeled data are predicted starting from differently labeled data and network structure) is applied to predict the gene function. GeneMANIA has been applied in several studies (e.g. O'Roak et al. (2012); Tkach et al. (2012); Giudice et al. (2014); Karki et al. (2018); Sepulcre et al. (2018)).

Differently from above methods, EPU (Ensemble Positive Unlabeled learning) (Yang et al., 2014) uses a supervised learning method, that falls in the class of Positive-Unlabeled learning method, for disease gene identification by integrating multiple biological data sources (PPI, gene expression data, Gene Ontology, Phenotype-gene association data and Phenotype similarity network). ND is applied on three biological networks (Gene Expression network, PPI network, Gene ontology similarity network) to obtain weights for unlabelled genes (not associated with disease). The resulting three collections of ND scores are combined into a set of integrated scores using, for each gene, the mean of its three ND scores (ND-first). These integrated scores are used to train three machine-learned prediction models (Weighted-KNN, Weighted-Naïve Bayes, Weighted-SVM) and their results are integrated by an ensemble learning algorithm.

## Discussion

ND based approaches have been proposed to solve several problems in biological data analysis, including data integration. These methods analyse multiple collections of scores derived from different omics assays in combination with molecular networks or similarity networks, and apply ND on such networks. The main applications include: gene function prediction; gene prioritization; identification of gene modules and molecular pathways; disease subtyping; and prediction of an outcome. In all these applications, ND is a tool to transform one or more initial vectors of scores into vectors that reflect the network proximity between network nodes on which the scores are mapped. This data transformation is exploited for different purposes, such as: embedding a molecular interaction data into omics datasets; amplifying associations between the studied variables; missing value imputation; enabling comparisons among different data types; highlighting network regions enriched in multiple types of scores; and studying molecular profiles at patient-level scale.

ND processes, which can be brought back to four classes, require the tuning of a parameter (*k* or α) that controls the diffusion process reach or the relative importance of topology and input scores. In many cases, the issue about tuning of such parameter has been solved showing that the performance of the proposed integrative method is robust to small variations of the parameter. A dependency between the optimal value and the network in use has been suggested (Hofree et al., 2013).

Most methods apply ND to transform a series of input score collections to get as many collections of ND scores—in which the network topology is embedded—and, subsequently, combine the ND scores: we referred to these methods as ND-first. The combination of a series of ND scores for the same variable (e.g. a gene) is performed with simple mathematical operators, such as the mean or the minimum, or with more elaborated techniques, such as non-negative matrix factorization and support vector machines. ND scores may require a step of transformation, such as normalization, to enable the direct comparison between scores at different scale [e.g. Hofree et al. (2013)], or ranking, to work on the relative importance rather than absolute values [(e.g. Ma et al. (2014); Shi et al. (2016))]. Other integrative methods, firstly integrate multiple data types, then use ND: we referred to these methods as *ND-after*. In these methods, ND is one of the last steps that lead to the final output. A third class of methods perform ND simultaneously with the integrative step (*ND-during*). The class of simultaneous diffusion approaches is very promising as it encodes the diffusion processes on multi-layer networks (Aleta and Moreno, 2019). In principle, simultaneous diffusion allows to extend the classical analysis of multi-omics data on complex networks. For instance, in the case of heterogeneous networks, layer-specific nodes bring an indirect contribution to the ND scores on each other layer. Such an output is not possible neither in *ND-first* nor in *ND-after* approaches. *ND-after* integrative approaches build an aggregate network encoding weighted or unweighted aggregate links; such an aggregate network is therefore algebraically put together, independently from the diffusion process. The same considerations hold for *ND-first* approaches, but such integration issues are addressed once the ND is performed on each layer separately. Therefore, *ND-after* and *ND-first* approaches could be very informative about a specific biological analysis but they present an intrinsic lack of scalability, as the way in which properly combine and weigh networks (before or after ND) strongly depends on the biological context. Conversely, an *ND-during* (simultaneous) approach maintains the available biological information and avoids additional data manipulations before and after the application of the diffusive algorithm. However, simultaneous approaches may introduce computational issues as omics data size and number of layers increase.

Most of the approaches do not assess the statistical significance of ND scores. In several works it was proposed to use empirical *p* values (Bersanelli et al., 2016b), which provide also the benefit of mitigating the over-estimation of hub importance. In a recent work, the calculation of empirical *p* values using degree-normalized random seeds was shown to be more accurate, but computationally more demanding, than random seeds (Biran et al., 2019).

A specific combination of omics (e.g. gene mutations and gene expression changes) and a quite specific formulation of the problem is often required. While this specificity offers advantages within the domain of the original problem, it also poses constraints to applicability and further extension. Furthermore, efforts are still required to develop methods that combine more than two omics.

A relevant issue is the reliability of interactomes. The problem of defining a reference human interactome is open in molecular biology as well as the problem of quantifying the reliability of such cell-scale reconstructions, because experimental technologies currently used to detect interactions involves a series of issues (Luck et al., 2017). Therefore, a careful network selection must be made by users based on the research questions they wish to address. Further, some methods take into account the directions of interactions in their algorithms, but cell-scale reconstructions do not provide information about “the direction” of the interaction, which requires a deeper understanding of the mechanistic relation between the two interacting partners. Modelling this information is not trivial and usually comes at the cost of a relevant reduction of coverage in terms of genes that can be analysed.

ND has become a popular tool in integrative analyses. The trends of recent works suggest that it will continue to be used and further refined as demands relative to new data types arise. For example, recent works apply ND to single cell data analysis, mainly to impute missing expression data (Van Dijk et al., 2018; Ye et al., 2019).

## Author Contributions

ND and EM conceived the study. ND, EM, and MB performed literature search and wrote the manuscript. ND drafted figures and tables. EM and LM critically reviewed the manuscript. All authors approved the final manuscript.

## Funding

This research was funded by: European Union's Horizon 2020 research and innovation programme, grant GEMMA 825033; Italian Ministry of Education, University and Research, project INTEROMICS PB05 and project BBMRI-it n. K75; Fondazione Regionale per la Ricerca Biomedica (Regione Lombardia), project LYRA 2015-0010 and project FindingMS ERAPERMED2018-233 GA 779282.

## Conflict of Interest

The authors declare that the research was conducted in the absence of any commercial or financial relationships that could be construed as a potential conflict of interest.
